# Resistance decay in individuals after antibiotic exposure in primary care: a systematic review and meta-analysis

**DOI:** 10.1186/s12916-018-1109-4

**Published:** 2018-08-07

**Authors:** Mina Bakhit, Tammy Hoffmann, Anna Mae Scott, Elaine Beller, John Rathbone, Chris Del Mar

**Affiliations:** Centre for Research in Evidence-Based Practice (CREBP), Faculty of Health Sciences and Medicine, Bond University, Gold Coast, QLD 4229 Australia

## Abstract

**Background:**

Antibiotic resistance is an urgent global problem, but reversibility is poorly understood. We examined the development and decay of bacterial resistance in community patients after antibiotic use.

**Methods:**

This was a systematic review and meta-analysis. PubMed, EMBASE and CENTRAL (from inception to May 2017) were searched, with forward and backward citation searches of the identified studies. We contacted authors whose data were unclear, and of abstract-only reports, for further information. We considered controlled or times-series studies of patients in the community who were given antibiotics and where the subsequent prevalence of resistant bacteria was measured. Two authors extracted risk of bias and data. The meta-analysis used a fixed-effects model.

**Results:**

Of 24,492 articles screened, five controlled and 20 time-series studies (total 16,353 children and 1461 adults) were eligible.

Resistance in *Streptococcus pneumoniae* initially increased fourfold after penicillin-class antibiotic exposure [odds ratio (OR) 4.2, 95% confidence interval (CI) 3.5–5.4], but this fell after 1 month (OR 1.7, 95% CI 1.3–2.1). After cephalosporin-class antibiotics, resistance increased (OR 2.2, 95%CI 1.7-2.9); and fell to (OR 1.6, 95% CI 1.2-2.3) at 1 month. After macrolide-class antibiotics, resistance increased (OR 3.8, 95% CI 1.9–7.6) and persisted for 1 month (OR 5.2, 95% CI 2.6–10.3) and 3 months (OR 8.1, 95% CI 4.6–14.2, from controlled studies and OR 2.3, 95% CI 0.6–9.4, from time-series studies).

Resistance in *Haemophilus influenzae* after penicillins was not significantly increased (OR 1.3, 95% CI 0.9–1.9) initially but was at 1 month (OR 3.4, 95% CI 1.5–7.6), falling after 3 months (OR 1.0, 95% CI 0.5–2.2). Data were sparse for cephalosporins and macrolides.

Resistance in *Enterobacter* increased post-exposure (OR 3.2, 95% CI 0.9–10.8, from controlled studies and OR 7.1, 95% CI 4.2–12, from time-series studies], but was lower after 1 month (OR 1.8, 95% CI 0.9–3.6).

**Conclusions:**

Resistance generally increased soon after antibiotic use. For some antibiotic classes and bacteria, it partially diminished after 1 and 3 months, but longer-term data are lacking and urgently needed.

**Trial registration:**

PROSPERO CRD42015025499.

## Background

The discovery of penicillin in the mid-20th century heralded the antibiotic era [1, 2] and contributed significantly to a decrease in the rates of morbidity and mortality that had been caused by previously life-threatening infections [3, 4]. However, antibiotic resistance emerged shortly afterwards [5]. This drove the discovery of new antibiotics [4]. However, the development of new antibiotics is no longer keeping up with resistance [6] and we now face the threat of a post-antibiotic era [7–9].

Antibiotic resistance is generated by its use [7]. One area of interest is the high use of antibiotics in primary care, particularly for the treatment of acute respiratory infections, for which there is very little or no benefit [10–14]. Yet many clinicians in primary care persist, believing that resistance is not their problem [15–17].

Systematic reviews suggest that prescribing antibiotics in primary care initially increases the prevalence of resistant bacteria in patients—more so in countries with higher prescribing rates [18]—but that they became less detectable in the microbiome after 12 months [19]. The return of the microbiome to antibiotic susceptibility is critical in encouraging a reduction of antibiotic use, which is being actively pursued in the primary-care community internationally to minimise antibiotic resistance. What remains unknown is the time this takes, and how it varies by antibiotic class and bacterium.

This information is important for informing public health messages, antibiotic resistance campaigns and clinician training. This systematic review aimed to identify and synthesise prospective studies that have examined the occurrence of bacterial resistance in community-based patients who were exposed to antibiotics, and to explore whether resistance decay varies by antibiotic class and bacterium.

## Methods

We initially planned simply to update a previous systematic review that had addressed resistance decay [19]. However, we were unable to replicate the search (since there were discrepancies in the numbers of studies found and differences in the eligible and included studies identified) and also realised that the time points were poorly discriminated, especially those from retrospective studies. The design of retrospective studies means that: (1) they can report only the time interval between antibiotic exposure and the isolation of resistant isolates at the end of the study, with no data in between; (2) details of the exposure antibiotic, such as type and dose, are often unknown and (3) there is often a selection bias towards patients with treatment failure. Accordingly, we undertook this review de novo.

This research was reported in line with the Preferred Reporting Items for Systematic Reviews and Meta Analyses (PRISMA) [20].

### Eligible study designs

Eligible studies compared antibiotic-exposed participants to controls (including randomised controlled trials or RCTs), or involved prospective repeat measure cohorts that reported the prevalence of resistant bacteria among patients, isolates or specimens (percentage of resistant isolates from each swab) over time. Retrospective studies were also identified as part of the same search process but will be reported separately. Case reports were ineligible.

### Eligible participants

We included studies of patients (or isolates from them), of any sex or age, symptomatic or asymptomatic, who were treated in the community or had community-acquired infections. Studies that included patients with hospital-associated infections, device-related infections and persistent infections were ineligible (Table 2 in the Appendix).

### Eligible types of antibiotic exposure

We included any study in which participants in the index group were exposed to a short antibiotic course (≤2 weeks), of any antibiotic class.

### Eligible comparison

Groups of participants who either did not use antibiotics (controls) or used them at different times were eligible as comparators.

### Outcomes

The primary outcome was the isolation of resistant bacteria at a pre-specified time point. Studies that did not report the duration between the last known antibiotic exposure and isolation of resistant bacteria, or did not report the before and after prevalences of resistant and susceptible isolates in studies comparing two antibiotic exposures, were excluded.

### Search and information sources

We searched PubMed, EMBASE and the Cochrane Central Register of Controlled Trials (CENTRAL) from inception until the first week of May 2017, using medical subject headings (MeSH) and keywords: ‘Drug Resistance’ AND ‘Anti-Bacterial Agents‘ AND ‘Primary Health Care‘ AND ‘Patients‘ with a detailed search strategy (Table 3 in the Appendix). Forward and backward citation searches identified additional relevant studies. We contacted authors whose data were unclear, and of abstract-only reports, for further information.

### Study selection

Two researchers (MB and JR) independently screened the titles and abstracts of search results using Endnote (version X8) and the Rayyan website for systematic reviews [21], and then the full texts of remaining studies for inclusion. A third reviewer (CDM or TH) resolved any disagreements.

### Data extraction

Two researchers (MB and AS) used a pre-specified and pre-piloted form to independently extract data on: study design, study duration, symptomatic or asymptomatic patients, age, recruitment location, total number of reported patients and isolates, methods of sampling, and collection of antibiotic exposure data and analysis. Disagreements were resolved by consensus or third author (CDM or TH).

### Assessment of risk of bias

Two researchers (MB and AS) independently evaluated the risk of bias, using the Cochrane Risk of Bias tool [22] for RCTs, or, for other study designs, items adapted from the Risk of Bias in Non-randomised Studies, Interventions (ROBINS-I) tool [23] (Box 1).

### Data analysis

We derived the odds of identifying resistance at different time points.

Some studies limited the denominator to participants carrying bacteria and others to total participants (those carrying bacteria or not). We included only data from participants carrying bacteria, which enabled comparisons, as we are interested in the burden of resistance on the community. We extracted incident cohort counts, where reported. If they were not, we manually calculated them from odds ratios (ORs). When resistance data were reported for more than one antibiotic, we analysed only resistance to the same antibiotic to which participants were exposed (to avoid duplication), and co-resistance data were extracted and reported in separate tables. Some studies reported resistance as ‘intermediate’ and ‘high’: we collapsed these into ‘resistant’.

We use the term ‘prospective repeated measures cohort studies’ to describe those that were randomised trials by design but in which the data were extracted from each arm of the trial separately without the benefit of randomisation. These were analysed with the cohort studies. The main study designs are detailed in Table 1.

**Table 1:** Characteristics of included studies

	Setting	Study design^a^				Participants				Total number of participants^b^	Age range		Sample site		Method of measuring resistance						Guidelines used				Sampling time points				
						S		AS																					
		RCT	COS - Nested in a RT	COS with a control group	COS	Adults	Children	Adults	Children		From	to	Respiratory	Gastrointestinal tract	Agar-dilution	Disk-diffusion	E Test	Paper disk testing	Broth-dilution method	ASS	NCCLS/CLSI	CASFM/EUCAST	German National S.	Not reported	Baseline	End of treatment	Days	Weeks	Months
Murray et al. [45] (Mexico 82)	?		✓			?				145	?			✓	✓	✓								✓	✓		14		
Huovinen et al. [42] (Finland 85)	hCC		✓			✓				97	16 y	64 y		✓	✓									✓	✓	✓			1
Brook, I [30] (USA 88)	PED			✓			✓			54	?		✓		?									✓	✓		7 to 10	5 to 7	3
Eliasson et al. [38] (Sweden 90)	hCC		✓				✓			150	0 m	10 y	✓					✓						✓	✓	✓		4	
Cohen et al. [32] (France 97)	PED		✓				✓			364	4 m	4.5 y	✓		✓			✓			✓				✓		2 to 6		
Dagan et al. [37] (Israel 98)	ER				✓		✓			120	3 m	3 y	✓			✓					✓				✓		4 & 5		
Dabernat et al. [36] (France 98)	PED & ENT		✓				✓			426	6 m	3 y	✓			✓					✓	✓			✓	✓			1
Cohen et al. [33] (France 99)	PED		✓				✓			513	4 m	2.5 y	✓		✓			✓			✓				✓		12 to 14		1
Chern et al. [26] (Nepal 99)	V	✓					✓		✓	122	1 y	10 y	✓				✓							✓	✓		14		
Ghaffar et al. [40] (USA 99)	PED		✓				✓		✓	160	6 m	6 y	✓				✓	✓			✓				✓			2	2
Morita et al. [44] (USA 00)	S				✓		✓		✓	300	?		✓				✓				✓				✓		17, 32		
Varon et al. [50] (France 00)	PED				✓		✓			705	3 m	3 y	✓		✓									✓	✓	✓	2 to 6		
Schrag et al. [48] (Dominican R. 01)	hOC		✓				✓			795	6 m	5 y	✓				✓				✓				✓		5, 10, 28		
Ghaffar et al. [41] (USA 02)	PED			✓			✓		✓	160	6 m	6 y	✓				✓	✓			✓				✓			2	2
Cremieux et al. [35] (France 03)	hCC		✓					✓		50	19 y	44 y	✓			✓								✓	✓	✓	14, 21,45		
Berg et al. [25] (Netherlands 04)	hOC	✓						✓		296	54 y	73 y	✓				✓				✓				✓	✓			2
Toltzis et al. [49] (USA 05)	PED		✓				✓			1009	3 m	7 y	✓				✓				✓				✓		3 to 5, 10 to 12		1
Gaynor et al. [39] (Nepal 05)	V		✓				✓		✓	444	12 m	7 y	✓						✓		✓								6
Lofmark et al. [43] (Sweden 06)	Vol			✓				✓		8	31 y	58 y		✓	✓					✓	✓				✓	✓		3	3, 6, 9, 12, 18, 24
Conradi et al. [34] (Spain 07)	hER				✓		✓			134	0 m	5 y	✓		✓						✓				✓	✓			1
Malhotra-Kumar et al. [27] (Belgium 07)	Vol	✓						✓		224	18 y	58 y	v							✓	✓				✓		8,28		
Chung et al. [31] (UK 07)	GP			✓			✓			119	6 m	12 y	✓				✓			✓	✓				✓			2, 12	
Raum et al. [47] (Germany 08)	GP				✓	✓				541	mean = 57.5			✓						✓			✓		✓		7,14		
Nord et al. [46] (USA 09)	OC		✓					✓		143	18 y	45 y	✓				✓				✓				✓	✓		2, 4, 6	
Skalet et al. [29] (Ethiopia 10)	V	✓							✓	10,778	12 m	10 y	✓				✓							✓					3
Malhotra-Kumar et al. [28] (Europe 16)	PC	✓				✓				102	20 y	81 y	✓							✓	✓				✓	✓	8, 14, 28, 42		6

*S* symptomatic, *AS* asymptomatic, *PED* paediatric clinics, *hOC* hospital outpatient clinic, GP general practices, *S* school, *OC* outpatient clinic, *hCC* health care centre, *hER* hospital emergency department, *V* villages, *Vol* volunteers, *PC* Primary care, *ASS* automated antimicrobial susceptibility testing systems, *RCT* randomised-controlled trials, *COS* prospective cohort study design, *RT* randomised trial, *NCCLS/CLSI* The Clinical and Laboratory Standards Institute, *CASFM*/*EUCAST* French/European Committee on Antimicrobial Susceptibility Testing

^a^Study design reported is based on the type of extracted data

^b^Numbers might be different from those included in the analysis

Resistance prevalence data can be compared at different time points in two ways, according to study design: a separate control group (methodologically more robust) or studies reporting before and after antibiotic exposure. We meta-analysed the two methods separately, but present them adjacently.

To facilitate comparisons, we collapsed the reported time periods after antibiotic exposure to pre-specified ranges: pre-exposure and from end of treatment (i.e. time 0): 0 to ≤1 week, >1 week to ≤1 month and >1 to ≤3 months. When the same study reported multiple resistance data that fell in the same pre-specified ranges, we chose the latest time point provided.

We undertook the meta-analysis using RevMan Version 5.3 [24], pooling Peto ORs from the end of treatment with a fixed-effects model to correct better for zero cell counts [22]. We assessed statistical heterogeneity among studies with a χ^2^ test (using *P* ≤ 0.05 for significant heterogeneity) and I^2^. Subgroup analyses were pre-specified by the time since last antibiotic exposure. We were not able to test for statistical differences between different times using either a statistical test for trend or a χ^2^ test for heterogeneity of the different time subgroups, as some studies provided data for different time points, but not all.

### Protocol and registration

The review protocol was registered on the PROSPERO database (CRD42015025499) at http://www.crd.york.ac.uk/PROSPERO/display_record.php?ID=CRD42015025499. Ethics approval was not required. A modification of the protocol was to clarify that studies that had reported resistant bacteria at the isolate level were also eligible.

## Results

### Study selection

**Fig. 1:**
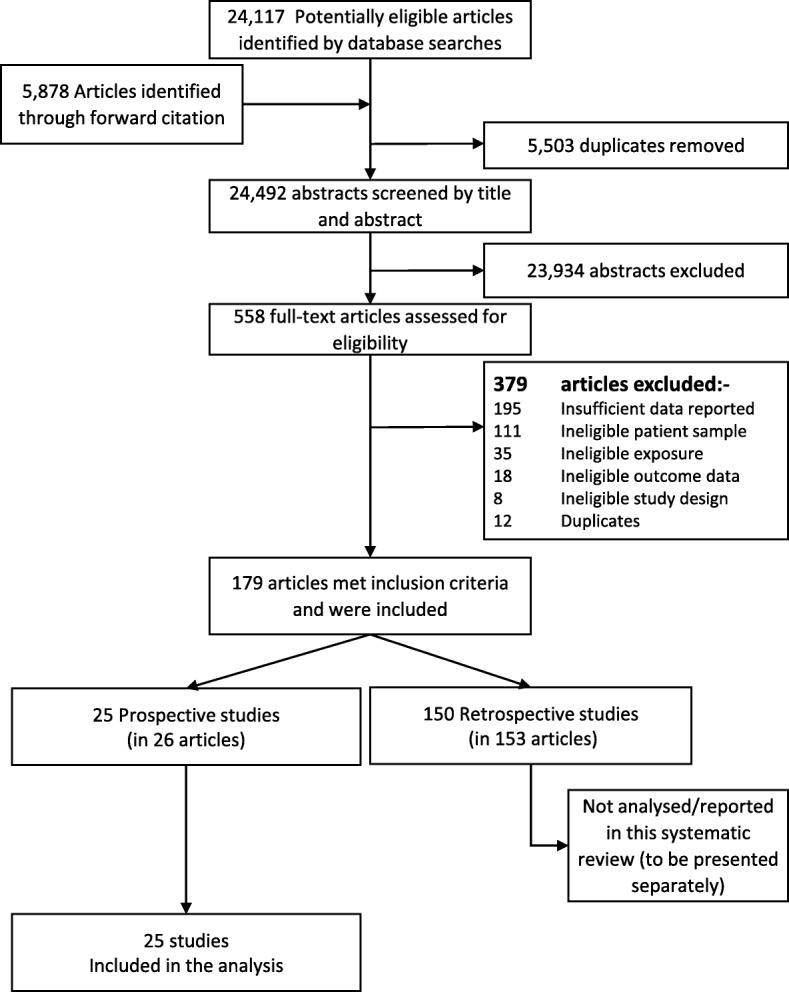
Study flow chart

Our search found 24,117 citations, supplemented by 5878 citations identified from forward and backward searches of references cited in included studies, which, after removing duplicates, left 24,492. Screening by title and abstract excluded 23,934, leaving 558 for which the full text was screened. After excluding 379 (Table 4 in the Appendix gives detailed reasons for exclusion), 179 eligible articles remained, of which 25 studies (in 26 articles) assessed the isolation of resistant bacteria prospectively. These were included in this review (Fig. 1).

### Study characteristics

Of the included studies, five were RCTs [25–29] and 20 were prospective cohort studies [30–50]. We report the study design here in relation to the outcome of resistance, although some studies were RCTs for the outcome of efficacy. Table 1 shows study characteristics. All but three [26, 29, 39] were conducted in one of the Organisation for Economic Co-operation and Development countries:

- 16 investigated children (total of 16,353) [26, 29–34, 36–41, 44, 45, 48–50] and 8 studied adults (total of 1461) [25, 27, 28, 35, 42, 43, 46, 47]
- 14 investigated symptomatic patients (12 with respiratory infections [28, 30–34, 36–38, 48–50], 1 with a urinary infection [42] and 1 with acute febrile illness [47])
- 6 involved asymptomatic participants [25, 27, 29, 35, 43, 46]
- 5 studies included both symptomatic and asymptomatic participants [26, 39, 41, 44, 45]

Twelve studies compared antibiotic exposure against a control or placebo [25–31, 39, 41, 43, 45, 47] and 13 were antibiotic comparison studies [32–38, 42, 44, 46, 48–50]. Antibiotics from the following classes were studied: penicillins (14) [28, 30–38, 41, 48–50], macrolides (12) [25–27, 29, 37, 39, 41, 44, 46, 49, 50], cephalosporin (8) [31–33, 36–38, 49, 50], sulphonamides and trimethoprim (2) [42, 45], quinolones (1) [46], lincomycin (1) [43] and ketolides (1) [35]. One study included any antibiotic [47].

### Risk of bias in studies and heterogeneity assessment

**Fig. 2:**
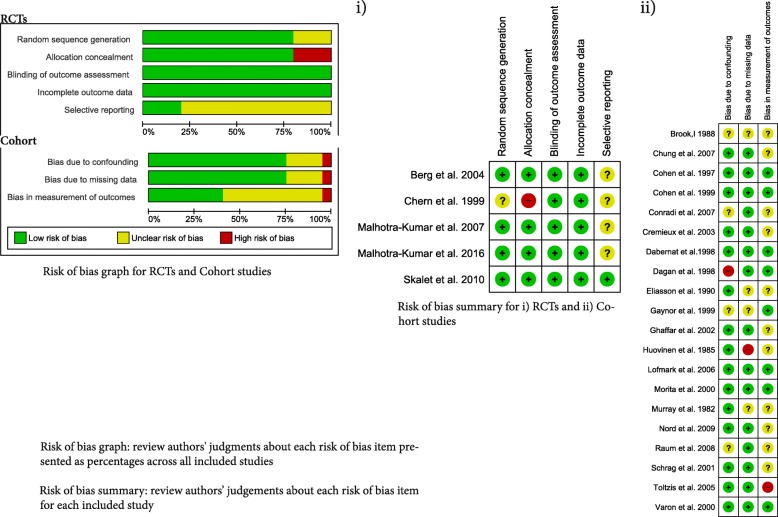
**a**. Risk of bias graph: review authors’ judgments about each risk of bias item presented as percentages across all included studies. **b.** Risk of bias summary: review authors’ judgements about each risk of bias item for each included study. RCT randomised controlled trial

**Fig. 3:**
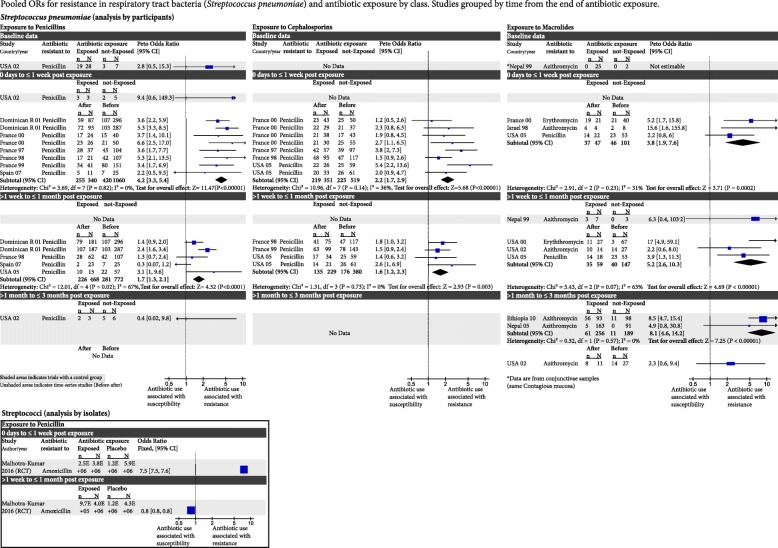
Pooled odds ratios for resistance in respiratory tract bacteria (*Streptococcus pneumoniae*) and antibiotic exposure by class. Studies grouped by time from the end of antibiotic exposure. CI confidence interval, df degrees of freedom, RCT randomised controlled trial

**Fig. 4:**
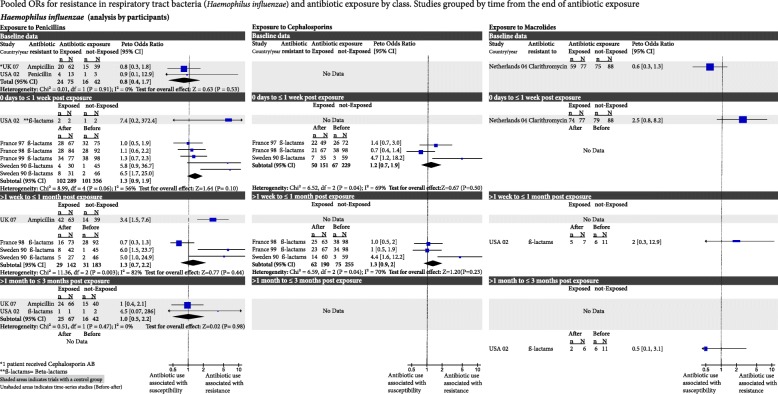
Pooled odds ratios for resistance in respiratory tract bacteria (*Haemophilus influenzae*) and antibiotic exposure by class. Studies grouped by time from the end of antibiotic exposure. CI confidence interval, df degrees of freedom

**Fig. 5:**
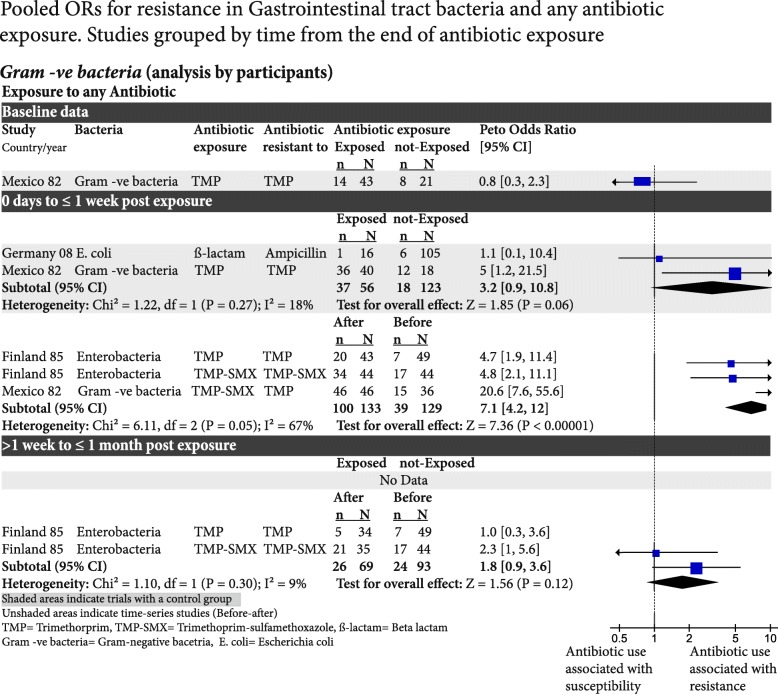
Pooled odds ratios for resistance in gastrointestinal tract bacteria and any antibiotic exposure. Studies grouped by time from the end of antibiotic exposure. -ve, negative, CI confidence interval, df degrees of freedom, SMX sulfamethoxazole, TMP trimethoprim

The risk of bias was assessed based on the study design for the outcome of resistance, not the original study design for the outcome of efficacy. The overall risk of bias was low, although bias due to selective reporting was uncertain for most RCTs because resistance was often not nominated as an outcome and there was an unclear risk of bias for the outcome measurement in the cohort studies (Fig. 2). We were not able to test for publication bias for the examined outcomes because of the very low number of studies in each funnel plot (Fig. 8 in the Appendix). There was considerable variation in the heterogeneity between studies, particularly for the cohort studies (Figs. 3, 4 and 5).

### Resistance in respiratory tract bacteria

Bacteria were isolated from the respiratory tract in 19 studies and from the conjunctiva in one study.

### *Streptococcus pneumoniae* and penicillin exposure

Penicillin-resistant *Streptococcus pneumoniae* were studied in only one controlled study (with 35 participants). Before exposure to penicillin, resistance was not significantly different between the group of patients subsequently exposed and those not exposed [OR 2.8, 95% confidence interval (CI) 0.5–15.3]. After exposure, the OR of resistance in those exposed was 9.4 (95% CI 0.6–149.3). After 3 months, there was no longer a significant difference in resistance (OR 0.4, CI 0.02–9.8; Fig. 3).

There were more data from prospective repeated measures cohort studies that compared resistance rates before antibiotic exposure (baseline data) and after penicillin exposure after 1 week (0 to 7 days; 6 studies, 1060 participants and 8 antibiotic exposure groups) and after 1 month (>1 week to ≤1 month; 4 studies, 772 participants and 5 antibiotic exposure groups). After 1 week, resistance had increased significantly (OR 4.2, 95% CI 3.3–5.4). Thereafter, resistance had reduced after 1 month (OR 1.7, 95% CI 1.3–2.1; Fig. 3).

One RCT [28] investigated reported resistance in isolates (rather than individuals) after exposure to amoxicillin and its data are analysed separately. It found that the changes in resistance following amoxicillin exposure were short-lived, returning to normal by 1 month after the end of treatment (Fig. 3).

### *S. pneumoniae* and cephalosporin exposure

There were no RCTs. Four cohort studies (519 participants and 8 different antibiotic exposure groups) reported that resistance had increased at 1 week after exposure (OR 2.2, 95% CI 1.7–2.9), persisting after 1 month (OR 1.6, 95% CI 1.2–2.3; Fig. 3).

### *S. pneumoniae* and macrolide exposure

There were three controlled studies. After a month, one small study reported the OR of resistance was 6.3 (95% CI 0.4–103.2). In three studies (437 participants), it remained high (OR 8.1, 95% CI 4.6–14.2) at 3 months. An RCT [27] of isolates found that a single course of macrolide-class antibiotics caused increased resistance in the first week immediately after macrolide use, and resistance remained significantly higher than the placebo group for more than 3 months (data not shown).

Three cohort studies (101 participants and 3 different antibiotics) reported increased resistance at 1 week (OR 3.8, 95% CI 1.9–7.6). Three studies (147 participants and 3 different antibiotics) found that after 1 month, resistance was increased (OR 5.2, 95% CI 2.6–10.3). There were 3-month data from only one study (OR 2.3, 95% CI 0.6–9.4; Fig. 3).

### *Haemophilus influenzae* and penicillin exposure

Two RCTs (117 participants) found comparable resistance between groups before exposure to penicillin (OR 0.8, 95% CI 0.4–1.7). One week after exposure, resistance had increased non-significantly in one RCT (with only 4 participants; OR 7.4, 95% CI 0.2–374). Increased resistance persisted for 1 month in another RCT (102 participants; OR 3.4, 95% CI 1.5–7.6). At 3 months, in this study, resistance had returned to normal (OR 1.0, 95% CI 0.5–2.2).

In four cohort studies (356 participants and 5 different antibiotic exposure groups), resistance was not increased at 1 week (OR 1.3, 95% CI 0.9–1.9). In two of the four cohort studies (183 participants and 3 different antibiotic exposure groups), it remained not increased at 1 month (OR 1.3, 95% CI 0.7–2.2; Fig. 4).

### *H. influenzae* and cephalosporin exposure

There were no RCTs. Three cohort studies (229 participants and 3 different antibiotic exposure groups) found resistance had not increased at 1 week (OR 1.2, 95% CI 0.7–1.9) or at 1 month (255 participants; OR 1.3, 95% CI 0.9–2; Fig. 4).

### *H. influenzae* and macrolide exposure

One RCT (175 participants) reported data at two time points. Before exposure, resistance was not significantly different between groups (0.6, 95% CI 0.3–1.3) and directly after macrolide exposure, resistance had increased in the exposed group (OR 2.5, 95% CI 0.8–8.2). One cohort study also reported two time points. Resistance had increased after exposure at 1 month (OR 2.0, 95% CI 0.3–12.9) and it had decreased by 3 months (OR 0.5, 95% CI 0.1–3.1; Fig. 4).

### Resistance in other respiratory bacteria

**Fig. 6:**
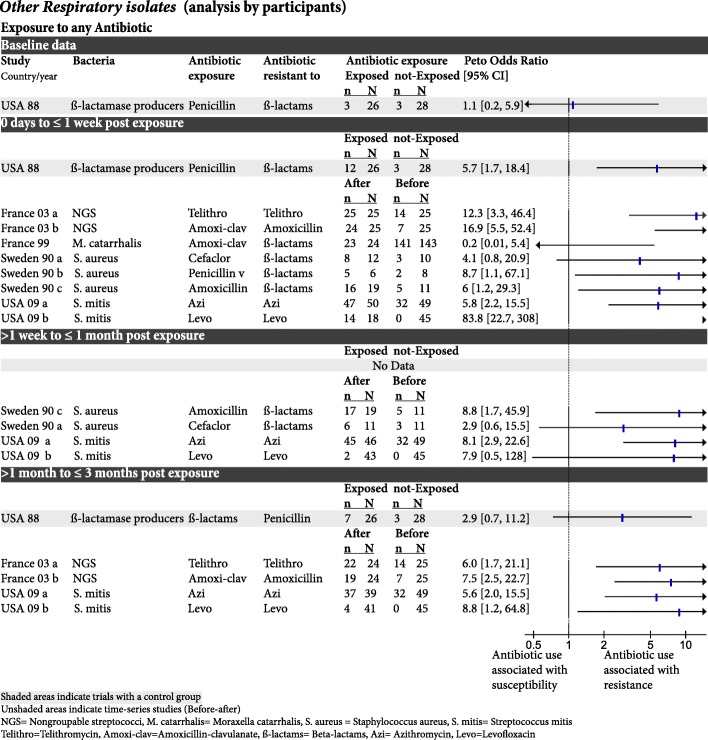
Odds ratio of resistance in other respiratory isolates post-exposure to different antibiotic classes. Shaded areas indicate trials with a control group. Unshaded areas indicate time-series studies (before and after). NGS non-groupable streptococci, *M. catarrhalis Moraxella catarrhalis*, *S. aureus Staphylococcus aureus*, *S. mitis Streptococcus mitis*, Telithro telithromycin, Amoxi-clav amoxicillin-clavulanate, ß-lactams beta-lactams, Azi azithromycin, Levo levofloxacin

The heterogeneity in five studies of resistance to *non-groupable streptococci*, *Moraxella catarrhalis*, *Staphylococcus aureus*, *beta-lactamase producers* and *Streptococcus mitis*, exposed to different antibiotic classes (penicillins, cephalosporins, macrolides, ketolides and quinolones), precluded meta-analysis. However, Fig. 6 in the Appendix shows a forest plot for the studies.

### Resistance in Gram-negative gastrointestinal tract bacteria to several antibiotics

*Trimethoprim and β-lactams exposure*: In one RCT (with 64 participants), before antibiotic exposure, the OR of isolating resistance was not significantly different at 0.8 (95% CI 0.3–2.3). Two controlled studies (with 179 participants) compared antibiotic exposure against a group with no exposure. It found that 1 week after antibiotic exposure, the OR of isolating resistant Gram-negative bacteria was 3.2 (95% CI 0.9–10.8; Fig. 5).

*Trimethoprim and trimethoprim-sulfamethoxazole exposure*: From two cohort studies (129 participants and 3 different antibiotic exposure groups) the OR of isolating antibiotic-resistant Enterobacteria was 7.1 (95% CI 4.2–12) at 1 week. In one study (with 93 participants and 2 different antibiotic exposures), the OR was 1.8 (95% CI 0.9–3.6) at 1 month (Fig. 5).

One RCT [43] investigated the consequences of a 1-week course of clindamycin on *Bacteroides* species using isolates rather than participants as the unit of analysis. It reported that the numbers of isolates returned to pre-treatment levels after 3 weeks in the exposed group. However, the isolates demonstrated qualitative changes to their diversity, and resistance genes remained 2 years later (data not shown).

### Co-resistance in participants in included studies

**Fig. 7:**
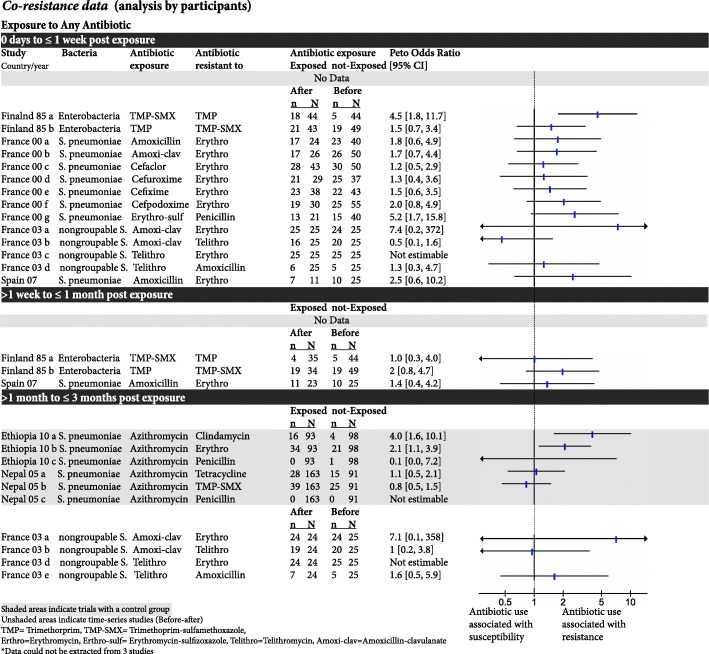
Co-resistance data reported among the included studies. Shaded areas indicate trials with a control group. Unshaded areas indicate time-series studies (before and after). TMP trimethorprim, TMP-SMX trimethoprim-sulfamethoxazole, Erthro erythromycin, Erthro-sulf erythromycin-sulfisoxazole, Telithro telithromycin, Amoxi-clav amoxicillin-clavulanate. *Data could not be extracted from three studies

**Fig. 8:**
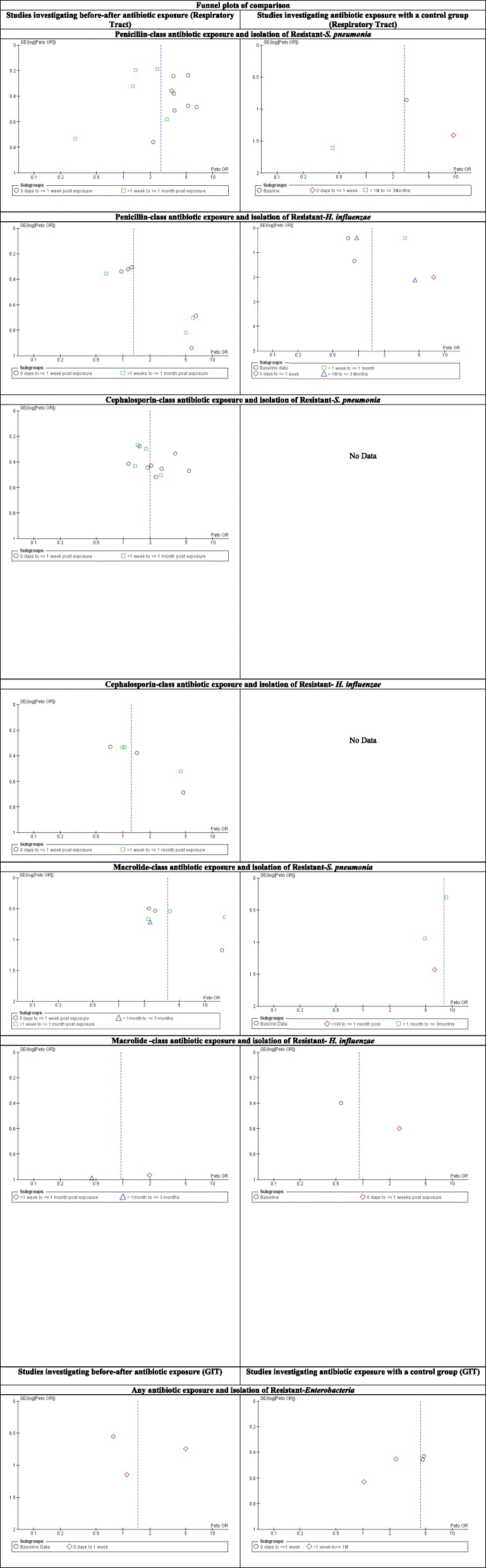
Funnel plots of comparison

Nine of the included studies reported selection for resistance to a different antibiotic than the exposure antibiotic (co-resistance). In respiratory isolates, 3 months after azithromycin exposure, the OR of isolating clindamycin-resistant *S. pneumoniae* (OR 4, 95% CI 1.6–10.1) and erythromycin-resistant *S. pneumoniae* (OR 2.1, 95% CI 1.1–3.9) was significantly higher between exposed and unexposed groups. In gastrointestinal tract Enterobacteria, there was a significant increase in the odds of isolating trimethoprim-resistant bacteria immediately after exposure to trimethoprim/sulfamethoxazole (OR 4.5, 95% CI 1.8–11.7; Fig. 7 in the Appendix).

## Discussion

Our systematic review found that antibiotic resistance in either the respiratory or gastrointestinal tracts of people in the community increased immediately after treatment with any of the antibiotics studied. This generally decayed over the next month, particularly in *S. pneumoniae* isolates treated with penicillins. The effect of cephalosporins on resistance was less pronounced at 1 week but persisted for at least for a month. After macrolide exposure, resistance persisted for at least 3 months. The paucity of controlled studies means there is some uncertainty around the estimates of the rate of decay of resistance in the macrolides.

There was no significant difference in isolation of resistant *H. influenzae* following penicillin or cephalosporin exposure. For macrolides, there were not enough data to examine this. For Gram-negative bacteria in the gastrointestinal tract, resistant bacteria were detectible 1 month after antibiotic exposure, decaying from immediately after exposure.

Antibiotic resistance may well predate the human exploitation of antibiotics [51]. Our data show that baseline antibiotic resistance increases after antibiotic use. The mechanism by which this happens includes selection of bacteria with the pre-existing gene and the acquisition of the resistance gene from other organisms in the microbiome. Similar mechanisms may be operating in the reversal of resistance when antibiotics disappear from the host environment.

This review, with its more up-to-date collection of studies, more rigorously collected data (from only prospective studies) and more precise time frames (which avoid the uncertainty implicit in time-until periods dictated by retrospective designs), confirms the broad finding of previous systematic reviews that antibiotic exposure results in resistance [18, 19].

It has been reported previously that isolation of resistant isolates was strongest in the month directly after exposure and remained detectable for up to 12 months [19]. However, our review provides better and more nuanced estimates of the time to decay of antibiotic resistance after exposure, with faster decays than previously reported. In addition, we show that the time frame may vary according to antibiotic class and bacteria, notwithstanding the limitations of the primary evidence.

Our search strategy was systematic and transparent, and found studies that had not been found in the earlier review of resistance decay [19]. Our review also provides a higher level of rigour by excluding studies at high risk of bias due to confounding variables (such as hospitalisation, device-associated infections and persistent infections) and by being careful to align the time periods after antibiotic exposure (as subgroup analyses) among the included studies to enable better comparisons.

There are several limitations of this review. First, the unadjusted status of the ORs we extracted, rather than simply importing study authors’ adjustments of some confounders, threatens to introduce bias from those confounders. There are potentially many other confounders. For example, resistance can be acquired through contact with other individuals rather than direct antibiotic exposure, groups within the included studies may have different baseline risks for resistance, resistance sampling was not standardised and indications for antibiotic exposure and bacterial load (likely to differ between symptomatic and asymptomatic participants, who might be the only carriers of resistance in their microbiome, or between children and adults) might affect the development of resistance. However, the crude ORs reported differ little from the adjusted values.

We were not able to investigate any effect of dose or duration of the antibiotic exposure on resistance. The quality of how resistance data were analysed and reported was poor in some studies, and some authors did not respond to our requests to clarify aspects of their methods and data, which contributes to the uncertainty of the review’s estimates. This could be because reporting of resistance was not the primary objective in most of the included studies. Finally, resistance was reported in most studies as the proportion of resistant isolates, which does not take account of the changes in overall bacterial population, which is likely to decrease from the antibiotic effect. Consequently, a rise in the resistance proportion might disguise a decrease in the absolute numbers of resistant bacteria.

Urgently needed is further research with high-quality placebo-controlled trials that measure the numbers of resistant and susceptible isolates and enable comparisons of antibiotic dose, duration and class against different bacteria.

## Conclusions

Antibiotic use increases the consequent isolation of bacterial resistance in individuals. The odds of resistance developing and the time of return to bacterial susceptibility may vary by antibiotic class. It appears that decay after exposure to antibiotics may be faster than previously reported [19] for penicillins against respiratory *S. pneumoniae*, and perhaps *H. influenzae*, although this may not be true for other antibiotics such as macrolides, where resistance might persist longer. This may be another factor for clinicians to consider when choosing an antibiotic, especially for minor infections. More primary research focussing on resistance development and decay is needed to further inform clinical decisions and public health policies.

## Box 1 Items adopted from the ROBINS-I tool for the cohort studies in this review

• Bias due to confounding:

- Confounding factors were adjusted in the analysis (low risk)

- Confounding factors were measured and showed balance (low risk)

- Randomised comparison (low risk)

• Bias due to missing data (follow-up data):

- Bias that arises when a later follow-up is missing for individuals initially included (low risk <20%)

• Bias in measurement of outcomes (who measured resistance):

- Independent lab (low risk)

- Independent technician (low risk)

- Study researchers (high risk)

## Appendix

**Table 2:** Elaboration on the inclusion and exclusion criteria

	Inclusion criteria	Exclusion criteria	Rationale for exclusion criteria
Population	Symptomatic and asymptomatic patients (healthy people)	Hospitalised patients with infections >48 h after admission	Increased risk of colonisation with drug-resistant bacteria from the hospital environment
	Hospitalised patients with a community infection (<48 h from admission)	Patients with post-surgery infections	
		Burn-associated infections	
		Sample of health-care workers, medical or nursing students with medical rotations	
		ICU patients referred from hospital wards or patients with central-line-associated bloodstream infections	High probability that these patients are infected with resistant bacteria
		Patients with device-related infections (catheter, implants, dialysis-associated infections or ventilation-associated infections)	Devices are more prone to infection with resistant bacteria
		Patients with persistent diseases (*tuberculosis, H. pylori,* syphilis, *Pseudomonas aeruginosa*, *Mycobacterium leprae* or *Salmonella typhi*)	Asymptomatic infections that remains undetected for a long duration; these require prolonged antibiotic treatments and it is considered treatment failure if the bacterium is isolated after treatment
		>50% of the sample are immunocompromised patients	Infections due to opportunistic bacteria that normally do not cause infections
		Patients with cystic fibrosis or bronchiectasis and cancer patients	Comorbidities that increase the risk of infection
Intervention	Any antibiotic exposure for any infection <14 days (prospective or retrospective)	Long-term antibiotic treatment >2 continuous weeks	Higher probability of killing susceptible organisms and increased risk of carriage of resistant isolates
Control/comparator	Patients without antibiotic exposure		
	Patients with a different antibiotic exposure, dose, frequency or route of administration	If there are no before and after measurements of resistance	
Outcome	Prevalence of resistance in exposed and unexposed patients	If there are no before and after measurements of resistance	
		Duplicate isolate reporting	
Time	Time between antibiotic exposure and isolation of resistant organisms	Studies were excluded if there were no data available on the last known antibiotic exposure	
Setting	Primary care		
	General practices		
	Outpatient clinics		
	Paediatric clinics		
	Emergency department		

*ICU* intensive care unit

**Table 3:** Search strategy

PubMed	
(‘Drug Resistance‘[Mesh] OR Resistance[tiab] OR Resistant[tiab] OR Multiresistant[tiab]) AND (‘Anti-Bacterial Agents‘[Mesh] OR ‘Macrolides‘[Mesh] OR ‘beta-Lactams‘[Mesh] OR Antibacterial[tiab] OR Antibacterials[tiab] OR Antibiotics[tiab] OR Antibiotic[tiab] OR Macrolides[tiab] OR Macrolide[tiab] OR beta-Lactams[tiab] OR Antimicrobial[tiab] OR Antimicrobials[tiab] OR Penicillin[tiab] OR Methicillin[tiab] OR ampicillin[tiab] OR azithromycin[tiab] OR Cephalexin[tiab]) AND (‘Population Surveillance‘[Mesh] OR ‘Primary Health Care‘[Mesh] OR ‘Ambulatory Care‘[Mesh] OR ‘Outpatients‘[Mesh] OR ‘Community-Acquired Infections‘[Mesh] OR ‘Demography‘[Mesh] OR ‘Carrier State‘[Mesh] OR ‘Endemic Diseases‘[Mesh] OR ‘Primary care’[tiab] OR ‘Primary healthcare’[tiab] OR ‘Family practice’[tiab] OR ‘General practice’[tiab] OR Ambulatory[tiab] OR Outpatients[tiab] OR Outpatient[tiab] OR Community[tiab] OR Communities[tiab] OR Surveillance[tiab] OR Carrier[tiab] OR Carriage[tiab] OR Area[tiab] OR Areas[tiab] OR Region[tiab] OR Regions[tiab] OR Demographic[tiab]) AND (‘Drug Prescriptions‘[Mesh] OR ‘Prescriptions‘[Mesh] OR ‘therapeutic use’[sh] OR Prescriptions[tiab] OR Prescription[tiab] OR Prescribing[tiab] OR Prescribe[tiab] OR Prescribed[tiab] OR Consumption[tiab] OR Courses[tiab] OR Course[tiab] OR Programme[tiab] OR Programmes[tiab] OR Dose[tiab] OR Doses[tiab] OR Exposure[tiab] OR Isolates[tiab] OR Isolated[tiab] OR Risk[ti]) AND (‘Patients‘[Mesh] OR ‘Drug therapy’[sh] OR ‘Drug effects’[sh] OR Microbiology[sh] OR Treatment[tiab] OR Patient[tiab] OR Patients[tiab] OR Patient’s[tiab]) AND (‘Randomised Controlled Trial’[pt] OR ‘Controlled Clinical Trial’[pt] OR ‘Epidemiologic Studies‘[Mesh] OR Randomly[tiab] OR Randomised[tiab] OR Randomised[tiab] OR Group[tiab] OR Groups[tiab] OR Control[tiab] OR Controlled[tiab] OR Case[tiab] OR Cases[tiab] OR Multicenter OR Center[tiab] OR Centre[tiab] OR Trial[tiab] OR Trials[tiab] OR Compare[tiab] OR Compared[tiab] OR Comparison[tiab] OR Cohort[tiab] OR Observed[tiab] OR Observational[tiab] OR Questionnaires[tiab] OR Questionnaires[tiab] OR Frequency[tiab] OR Frequencies[tiab] OR Baseline[tiab] OR Modelling[tiab]) NOT (‘Hospitals‘[Mesh] OR ‘Inpatients’[Mesh] OR ‘Cross Infection‘[Mesh] OR Hospitals[ti] OR Hospital[ti] OR Inpatients[tiab] OR Inpatient[tiab] OR ‘Cross infection’[tiab] OR ‘Cross infections’[tiab] OR ‘Hospital acquired’[tiab] OR ‘Hospital infection‘[tiab] OR ‘Hospital infections‘[tiab] OR Animal[tiab] OR Animals[tiab]) NOT (Review[pt] OR Meta Analysis[pt] OR News[pt] OR Comment[pt] OR Editorial[pt] OR Letter[pt] OR comment on[ti] OR systematic review[ti] or literature review[ti]) NOT (Animals[Mesh] not (Animals[Mesh] and Humans[Mesh]))	
CENTRAL (Cochrane)	
([mh ‘Drug Resistance‘] OR Resistance:ti,ab OR Resistant:ti,ab OR Multiresistant:ti,ab) AND ([mh ‘Anti-Bacterial Agents‘] OR [mh ‘Macrolides‘] OR [mh ‘beta-Lactams‘] OR Antibacterial:ti,ab OR Antibacterials:ti,ab OR Antibiotics:ti,ab OR Antibiotic:ti,ab OR Macrolides:ti,ab OR Macrolide:ti,ab OR beta-Lactams:ti,ab OR Antimicrobial:ti,ab OR Antimicrobials:ti,ab OR Penicillin:ti,ab OR Methicillin:ti,ab OR ampicillin:ti,ab OR azithromycin:ti,ab OR Cephalexin:ti,ab) AND ([mh ‘Population Surveillance‘] OR [mh ‘Primary Health Care‘] OR [mh ‘Ambulatory Care‘] OR [mh ‘Outpatients‘] OR [mh ‘Community-Acquired Infections‘] OR [mh ‘Demography‘] OR [mh ‘Carrier State‘] OR [mh ‘Endemic Diseases‘] OR ‘Primary care’:ti,ab OR ‘Primary healthcare’:ti,ab OR ‘Family practice’:ti,ab OR ‘General practice’:ti,ab OR Ambulatory:ti,ab OR Outpatients:ti,ab OR Outpatient:ti,ab OR Community:ti,ab OR Communities:ti,ab OR Surveillance:ti,ab OR Carrier:ti,ab OR Carriage:ti,ab OR Area:ti,ab OR Areas:ti,ab OR Region:ti,ab OR Regions:ti,ab OR Demographic:ti,ab) AND ([mh ‘Drug Prescriptions‘] OR [mh ‘Prescriptions‘] OR ‘therapeutic use‘:kw OR Prescriptions:ti,ab OR Prescription:ti,ab OR Prescribing:ti,ab OR Prescribe:ti,ab OR Prescribed:ti,ab OR Consumption:ti,ab OR Courses:ti,ab OR Course:ti,ab OR Programme:ti,ab OR Programmes:ti,ab OR Dose:ti,ab OR Doses:ti,ab OR Exposure:ti,ab OR Isolates:ti,ab OR Isolated:ti,ab OR Risk:ti) AND ([mh ‘Patients‘] OR ‘Drug therapy‘:kw OR ‘Drug effects‘:kw OR Microbiology:kw OR Treatment:ti,ab OR Patient:ti,ab OR Patients:ti,ab OR Patient’s:ti,ab) NOT ([mh ‘Hospitals‘] OR [mh ‘Inpatients‘] OR [mh ‘Cross Infection‘] OR Hospitals:ti OR Hospital:ti OR Inpatients:ti,ab OR Inpatient:ti,ab OR ‘Cross infection‘:ti,ab OR ‘Cross infections‘:ti,ab OR ‘Hospital acquired‘:ti,ab OR ‘Hospital infection‘:ti,ab OR ‘Hospital infections‘:ti,ab OR Animal:ti,ab OR Animals:ti,ab) NOT ([mh Animals] not ([mh Animals] and [mh Humans]))	
EMBASE	
(‘Drug Resistance’/exp. OR Resistance:ti,ab OR Resistant:ti,ab OR Multiresistant:ti,ab) AND (‘antibiotic agent’/exp. OR ‘Macrolide’/exp. OR ‘beta lactam’/exp. OR Antibacterial:ti,ab OR Antibacterials:ti,ab OR Antibiotics:ti,ab OR Antibiotic:ti,ab OR Macrolides:ti,ab OR Macrolide:ti,ab OR beta-Lactams:ti,ab OR Antimicrobial:ti,ab OR Antimicrobials:ti,ab OR Penicillin:ti,ab OR Methicillin:ti,ab OR ampicillin:ti,ab OR azithromycin:ti,ab OR Cephalexin:ti,ab) AND (‘health survey’/exp. OR ‘Primary Health Care’/exp. OR ‘Ambulatory Care’/exp. OR ‘Outpatient’/exp. OR ‘Community-Acquired Infection’/exp. OR ‘Demography’/exp. OR ‘heterozygote’/exp. OR ‘Endemic Disease’/exp. OR ‘Primary care’:ti,ab OR ‘Primary healthcare’:ti,ab OR ‘Family practice’:ti,ab OR ‘General practice’:ti,ab OR Ambulatory:ti,ab OR Outpatients:ti,ab OR Outpatient:ti,ab OR Community:ti,ab OR Communities:ti,ab OR Surveillance:ti,ab OR Carrier:ti,ab OR Carriage:ti,ab OR Area:ti,ab OR Areas:ti,ab OR Region:ti,ab OR Regions:ti,ab OR Demographic:ti,ab) AND	

**Table 4:** Detailed reasons for exclusion

Insufficient data reported	No individual patient data reported, reporting only *P* value	62
	No data on the number of resistant isolates	49
	No data on the number of patients exposed to antibiotics	47
	Time between antibiotic exposure and isolation of resistance not reported	14
	Contacted authors and no response received or full text not received (for conference abstracts)	23
Ineligible participant criteria	Hospitalised patients >50% (or hospital-associated infections or inpatients)	89
	Patients with persistent infections, device-related infections or tract abnormalities	11
	Immunocompromised patients	5
	>50% nursing home residents	1
	Reporting gene mutations or in vitro resistant isolates	5
Ineligible exposure	Prolonged antibiotic exposure (>2 weeks of exposure)	33
	Pharmacokinetics of antibiotic exposure	2
Ineligible outcome data	No before and after outcome data in studies in which all patients received antibiotic treatment	11
	Mixed data between resistant and susceptible isolates or all patients have resistant isolates	7
Ineligible study design	Case series, case reports, reviews and reports	8
Duplicates		12
Total		379

## Abbreviations

CI: Confidence interval
*H. influenzae*: *Haemophilus influenzae*
MeSH: Medical subject headings
OR: Odds ratio
PRISMA: Preferred Reporting Items for Systematic Reviews and Meta Analyses
RCT: Randomised controlled trial
ROBINS-I: Risk of Bias in Non-randomised Studies, Interventions
*S. pneumoniae*: *Streptococcus pneumoniae*
